# Optimization of Deuteron Irradiation of ^176^Yb for Producing ^177^Lu of High Specific Activity Exceeding 3000 GBq/mg

**DOI:** 10.3390/molecules28166053

**Published:** 2023-08-14

**Authors:** Lin Shao

**Affiliations:** Department of Nuclear Engineering, Texas A&M University, College Station, TX 77843, USA; lshao@tamu.edu

**Keywords:** isotope production, theranostic radionuclide, accelerator, lutetium, cancer treatment, medical isotope

## Abstract

The irradiation of ^176^Yb with deuterons offers a promising pathway for the production of the theranostic radionuclide ^177^Lu. To optimize this process, calculations integrating deuteron transport, isotope production, and decay have been performed. In pure ^176^Yb, the undesired production of ^174g+m^Lu occurs at higher deuteron energies, corresponding to a distribution slightly shallower than that of ^177^Lu. Hence, ^174g+m^Lu can be effectively filtered out by employing either a low-energy deuteron beam or stacked foils. The utilization of stacked foils enables the production of ^177^Lu using a high-energy linear accelerator. Another unwanted isotope, ^176m^Lu, is produced roughly at the same depth as ^177^Lu, but its concentration can be significantly reduced by selecting an appropriate post-irradiation processing time, owing to its relatively short half-life. The modeling approach extended to the mapping of yields as a function of irradiation time and post-irradiation processing time. An optimized processing time window was identified. The study demonstrates that a high-energy deuteron beam can be employed to produce ^177^Lu with high specific activity exceeding 3000 GBq/mg. The effect of different purity levels (ranging from 98% to 100%) was also discussed. The impurity levels have a slight impact. The modeling demonstrates the feasibility of obtaining ^177^Lu with a specific activity > 3000 GBq/mg and radionuclidic purity > 99.5% when using a commercially available ^176^Yb target of 99.6% purity.

## 1. Introduction

Theranostics is an emerging approach that combines therapeutic and diagnostic elements for effective cancer treatment [1]. In this approach, radionuclides emitting low-energy gamma rays are used for diagnostic purposes, while those emitting charged particles such as beta rays and alpha particles are utilized for therapy. This fusion of diagnostics and therapy represents a significant advancement in personalized cancer treatment [2,3]. ^177^Lu has garnered considerable interest as a theranostic radionuclide due to its emission of beta rays with an energy of 134 keV and low-energy gamma rays at 208 keV [4,5]. Combining ^177^Lu with other therapeutic radionuclides, such as ^90^Y/^177^Lu and ^67^Cu/^177^Lu, has shown great promise in cancer treatment [6,7]. The efficacy of ^177^Lu in neuroendocrine tumors has been acknowledged by the US Food and Drug Administration (approved in 2018) and the European Medicines Agency (approved in 2017) [8].

The term “carrier-free” is used to describe radionuclides with the highest specific activity. This means that the final product has 100% isotopic abundance and is not contaminated with stable isotopes. ^177^Lu, which has a high specific activity, is particularly important in certain radiation therapies, although it may not be necessary for all types. For instance, in peptide receptor radionuclide therapy, the limited concentration of different cellular cognate receptors expressed on the tumor cell surface necessitates the use of ^177^Lu with high specific activity. 

Currently, the production of ^177^Lu relies primarily on reactors using two production routes [9,10]: the “direct” route and the “indirect” route. In the direct route, the ^176^Lu target is subjected to neutron irradiation via Lu(n,γ)176Lu177 reactions. In comparison, the indirect route involves the use of a ^176^Yb target via Yb(n,γ)176Yb177→Lu177 reactions. Each route has its advantages and disadvantages.

The direct route benefits from the high thermal neutron capture cross-sections of ^176^Lu, which is as high as 2090 barn [11,12]. However, a drawback of the direct route is the production of ^177m^Lu as an impurity of concern. ^177m^Lu has a relatively long half-life of 160.5 days, leading to an increasing ratio of ^177m^Lu/^177^Lu over time. This poses challenges in hospital preparations, as the presence of ^177m^Lu triggers concerns regarding radioactive waste management. Currently, the average specific activity of the direct route is approximately 740 to 1100 GBq/mg, which needs further improvement.

The indirect production route is based on the Yb(n,γ)176Yb177→Lu177 reactions. This route offers the advantage of producing high specific activity, approximately 2960 GBq/mg, as it does not generate ^177m^Lu as an impurity. Furthermore, the product obtained via the indirect route can be carrier-free. However, there are some disadvantages associated with the indirect production route. It has low production yields due to the low cross-section of ^176^Yb, which is only 2.5 barns. Additionally, the chemical properties of Yb and Lu are very similar, posing challenges in the separation of Yb and Lu [13]. For more information on the various methods under development for Yb/Lu separation, a comprehensive review can be found in reference [13].

Considering the anticipated high market demand, alternative approaches utilizing accelerators have been investigated. These methods involve the use of protons [14], deuterons [15,16,17,18], alpha particles [19], and electron beams [20]. Among various accelerator-based techniques, the most efficient method for producing ^177^Lu is via the irradiation of a pure Yb target with deuterium [21]. This method has the highest yield in comparison with other possible choices, including ^nat^Yb(d,x)^177^Lu, ^nat^Hf(p,x)^177^Lu, ^nat^Hf(d,x)^177^Lu, ^nat^Lu(p,x)^177^Lu, ^nat^Lu(d,x)^177^Lu, and ^nat^Yb(α,x)^177^Lu. 

Previous studies have modeled the utilization of deuteron irradiation on a Yb target for ^177^Lu production [22,23]. Kambali compared the production yields between (d,n) and (d,p) reactions [22]. Nagai et al. systematically modeled the activities and specific activities of ^177^Lu using deuteron beams of different energies and Yb targets of varying purities [23]. Both studies suggested the feasibility of the overall processes. The present study aims to accomplish two objectives: firstly, to assess the feasibility of producing ^177^Lu using a high-energy linear accelerator (LINAC); secondly, to optimize both the irradiation time and the post-irradiation processing time to attain the highest achievable specific activity.

LINAC is a unique type of accelerator that can achieve very high beam energy at a relatively low cost. However, both the ion species and beam energies are fixed characteristics determined by the beam design. In other words, if it is designed at high energy, it cannot be operated at low energies. A high-energy LINAC provides opportunities for isotope production that requires high threshold beam energies. Conversely, a high-energy beam may be less suitable for isotope production that necessitates low threshold energies. An example of such a high-energy accelerator is the LINAC, located in Denton, Texas. Originally manufactured as part of the superconducting supercollider project, it was designed to operate at a beam energy close to 70 MeV for isotope production. This LINAC utilized a design that originated from Los Alamos National Laboratory in the 1980s, specifically tailored for an energy range of 70 to 90 MeV for the nuclear medicine program at that time [24,25]. Therefore, exploring the applications of high-energy LINACs for isotope production holds commercial value. It is worthwhile to investigate the feasibility of utilizing high-energy accelerators as versatile instruments capable of accommodating a wide range of isotopes requiring thermal energy at different energy levels, both low and high. 

The current study utilized 80 MeV as an illustrative example of a high-energy LINAC. However, the proposed methodology is applicable to various high-energy LINACs, regardless of whether they operate at 80 MeV or not. The multiple foil target configurations, as proposed in the present study, can be adjusted based on the specific beam energy of any LINAC, making the approach versatile and not limited to Denton LINAC. For the same reason, the beam current was chosen to be typical of the Denton LINAC. Nevertheless, the obtained yields can be readily converted for other LINACs. Hence, the impact of this study extends to general high-energy LINACs and is not specific to the Denton LINAC.

## 2. Modeling Procedure

The modeling approach employed in this study consists of the following steps: (1) a Monte Carlo simulation code was utilized to determine the energy of deuterons at different penetration depths; (2) the localized energy is converted into localized isotope production using energy-dependent activation functions; (3) the effects of continuous ion bombardment (gain) and decay (loss) are calculated as functions of irradiation time and post-irradiation processing time; and (4) the amount of produced isotopes is integrated over the region of interest to calculate activity and specific activity.

**Figure 1:**
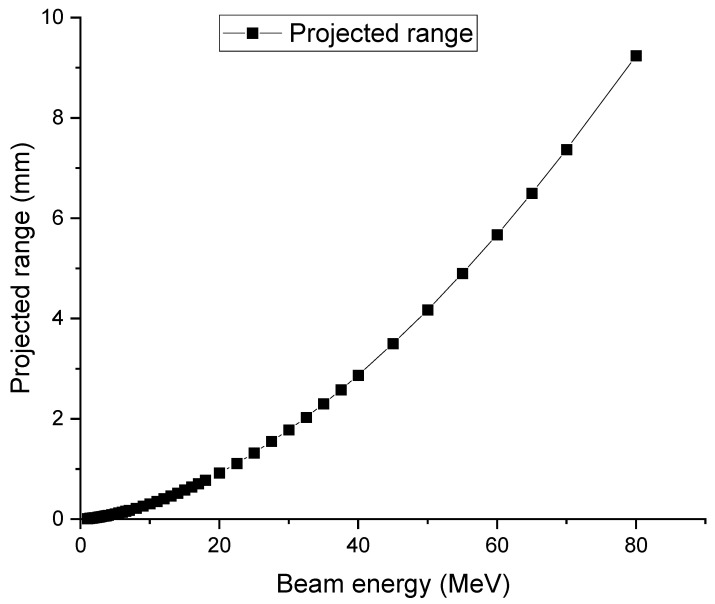
Projected ranges of deuteron as a function of deuteron incident energy in Yb.

**Figure 2:**
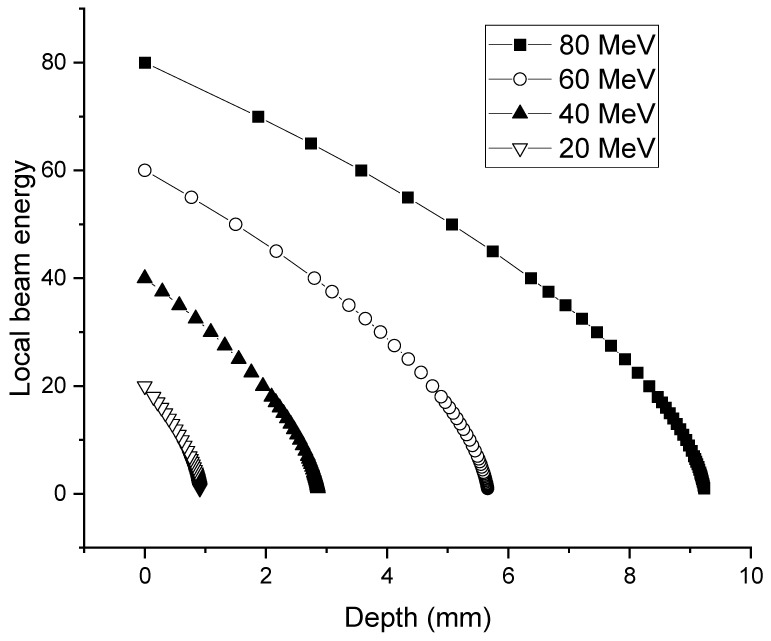
Local deuteron energy as a function of depth in Yb for incident energies of 20, 40, 60, and 80 MeV.

The Stopping and Range of Ions in Matter (SRIM) code has been widely used in materials science for irradiation studies [26]. However, SRIM does not directly provide detailed information about the local beam energy at different penetration depths as an output. Nevertheless, this information can be estimated reasonably well by analyzing the projected range of ions at various incident energies. Figure 1 plots the projected ranges of deuterons as a function of incident energy. For instance, at an incident energy of 80 MeV, the projected range of deuterium in Yb is approximately 9.2 mm. Conversely, at a lower energy of 60 MeV, the projected range reduces to 8 mm. This difference of 1 mm in range suggests that, in order for an 80 MeV beam to stop at 9.2 mm, its energy at a depth of 1 mm must be around 60 MeV. In other words, if we denote the projected range curve as *R(E)*, where *E* is the incident energy as a variable, then for a selected incident energy $E_{0}$ , the energy at a depth of $R\left(E_{0}\right)-R\left(E\right)$ from the surface is E . Figure 2 plots the local energy as a function of depth for different incident energies, calculated using the procedure described.

The utilization of Figure 2 to convert local beam energy into isotope production, based on energy-dependent cross-sections, assumes that straggling can be neglected. As a result, for a given incident energy, the energy values at each depth point are precise and have minimal uncertainty. This approximation holds true for protons and deuterons because their collisions with target atoms are primarily influenced by glancing angle collisions. Consequently, the ion trajectory remains a straight line for most of the penetration, except towards the very end, where low-energy collisions favor the creation of small damage cascades.

**Figure 3:**
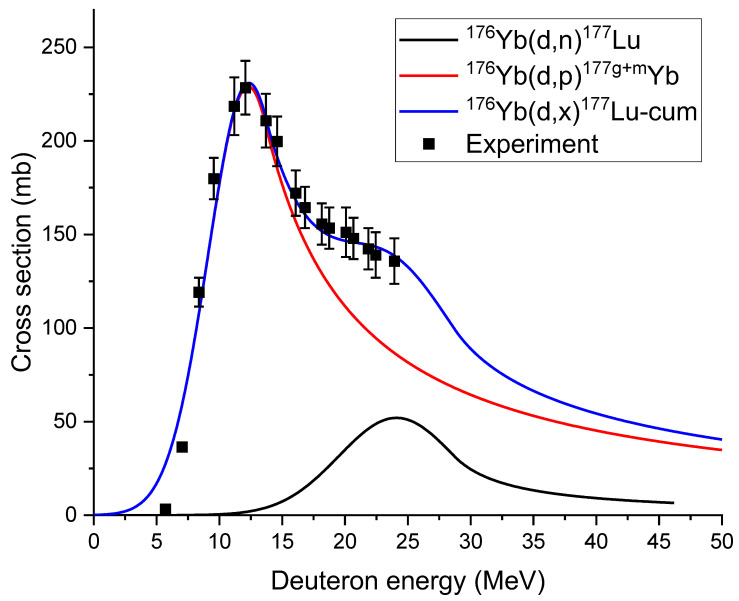
Cross-sections of producing Lu177 as a function of deuteron energy, from experiments [26] and fitting [23].

The available cross-section data for deuterium bombardment of Yb176 and other Yb isotopes are quite limited. Khandaker et al. conducted measurements on natural Yb and reported cross-section data for (d,x) reactions up to 24 MeV [27]. Nagai et al. fitted these cross-section data and experimentally validated the integrated yields [23]. Figure 3 plots the experimental activation functions for producing Lu177 in pure Yb176 [27], along with the fitted functions [23]. Two reactions result in the production of ^177^Lu. The first reaction is Yb(d,n)176Lu177 , and the second reaction is Yb(d,p)176Yb177g+m→Lu177 . The cross-section of the Yb(d,p)176Yb177g+m→Lu177 reaction is higher than that of the Yb(d,n)176Lu177 reaction. Yb177 undergoes β emission followed by gamma transitions in Lu177 , with a relatively short half-life of 1.88 h. Therefore, Lu177 can be approximated as the direct product of deuteron irradiation. It is important to note that the available experimental data is limited to energies up to 24 MeV. Further validation is required to assess the accuracy of the data at higher energies.

**Figure 4:**
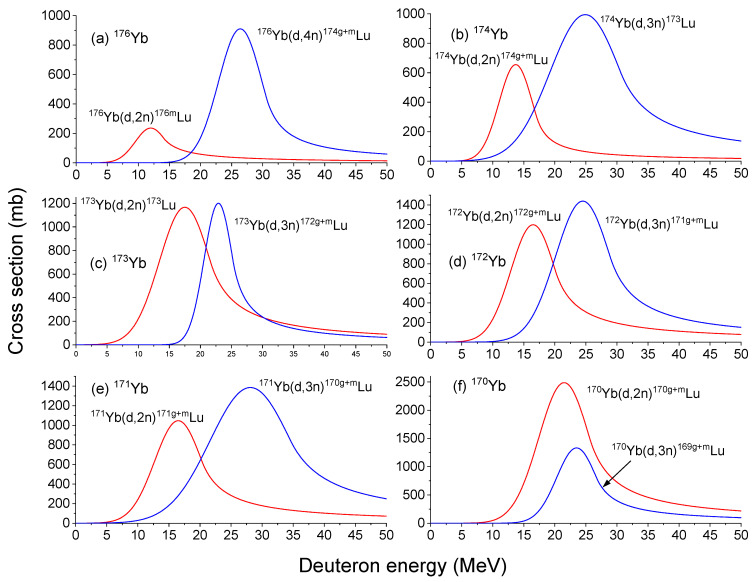
Cross-sections of various (d,x) reactions in deuteron-irradiated (**a**) Yb176 , (**b**) Yb174, (**c**) Yb173 , (**d**) Yb172 , (**e**) Yb171 , and (**f**) Yb170 , as a function of deuteron energy [23].

To maximize the specific activity of ^177^Lu, minimizing the production of other isotopes is crucial. This is why purified ^176^Yb is preferred over natural Yb. Natural Yb consists of seven stable isotopes: ^176^Yb, ^174^Yb, ^173^Yb, ^172^Yb, ^171^Yb, ^170^Yb, and ^168^Yb, with ^174^Yb being the most abundant at 31.8% of natural abundance. Figure 4a–f presents the fitted cross-sections for producing isotopes other than ^177^Lu in ^176^Yb, ^174^Yb, ^173^Yb, ^172^Yb, ^171^Yb, and ^170^Yb, respectively. ^168^Yb is not included due to its very low abundance (0.126%). It is observed that almost all cross-sections of (d,x) reactions are higher than that for producing ^177^Lu in ^176^Yb (Figure 3). Therefore, utilizing purified ^176^Yb instead of natural Yb is the most effective approach to avoid the generation of unwanted isotopes.

Table 1 summarizes Lu isotopes (only relevant ones are selected) and their half-lives and decay modes [28,29]. As for specific isotope products in Figure 4, ^173^Lu produced from the bombardment of ^174^Yb or ^173^Yb decays into ^173^Yb via electron capture (EC) with a half-life of 1.37 years. For ^172g+m^Lu produced from both ^173^Yb and ^172^Yb, ^172^Lu decays into ^172^Yb via β+ decay with a half-life of 6.7 days. ^172m^Lu decays into ^172^Lu via isomeric transition (IT) with a half-life of 3.7 min. For ^171g+m^Lu produced from ^172^Yb and ^171^Yb, ^171^Lu decays to ^171^Yb via b β^+^ decay with a half-life of 8.24 days. ^171m^Yb decays to ^171^Lu via IT decay with a half-life of 79 s. For ^170g+m^Lu produced from ^171^Yb and ^170^Yb, ^170^Lu undergoes β+ decay into ^170^Yb with a half-life of 2 days. ^170m^Lu decays rapidly into ^170^Lu via IT decay with a half-life of 670 milliseconds. For ^169^Lu produced from bombarding ^170^Yb, it decays into ^169^Yb with a half-life of 34 h.

Figure 4a is important in minimizing the presence of unwanted isotopes when using a purified ^176^Yb target. ^176m^Lu, in particular, undergoes β^−^ decay into ^176^Hf (99.9%) and EC decay into ^176^Yb (0.095%). With a relatively short half-life of 3.664 h, it is feasible to wait for a sufficient time period for ^176m^Lu to decay before initiating the chemical separation process. By optimizing the timing, the specific activity of ^177^Lu can be maximized while minimizing the presence of ^176m^Lu and its decay products.

^174g^Lu, with a half-life of 3.31 years, decays into ^174^Yb via β^+^ decay. The long half-life of ^174g^Lu poses a challenge in achieving a high specific activity of ^177^Lu. However, the reactions leading to its production start above 15 MeV and become significant above 20 MeV. Therefore, the concentration of ^174g^Lu can be minimized by optimizing the beam energy (E < 20 MeV). Regarding ^174m^Lu, it undergoes IT decay into ^174^Lu (99.38%) and EC decay into ^174^Yb (0.62%). Its relatively long half-life of 142 days presents concerns about quality control. However, since ^174m^Lu has a higher threshold energy at 20 MeV, its concentration can also be minimized via beam energy optimization (E < 20 MeV).

**Figure 5:**
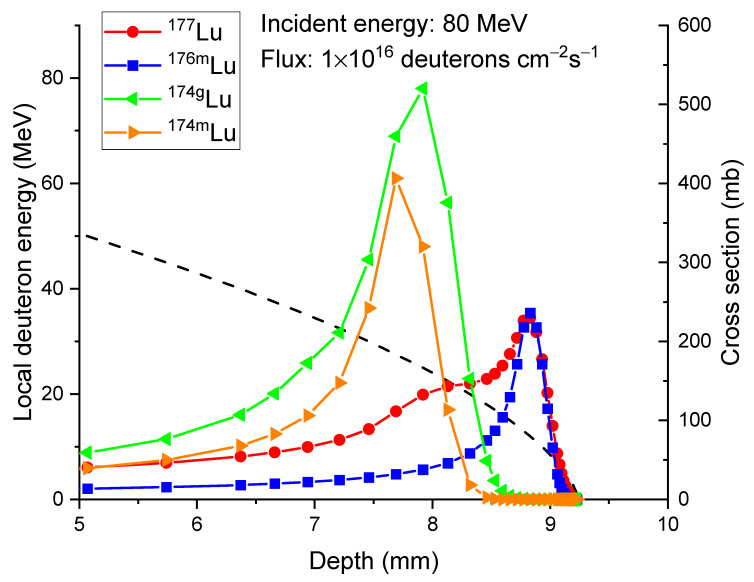
Local deuteron energy (dashed line) and cross-sections of producing ^177^Lu (red circle), ^176m^Lu (blue square), ^174g^Lu (green triangle), and ^174m^Lu (orange triangle), as a function of depth, for the case of 80 MeV deuteron irradiation of pure ^176^Yb.

The distinctive energy dependence of the production of ^177^Lu and other isotopes in pure ^176^Yb leads to the difference in their isotope distribution profiles, as shown in Figure 5 for the case of 80 MeV deuteron irradiation. The dashed line in Figure 5 represents the local deuteron energy as a function of penetration depth. The symbols represent the cross-section of producing ^177^Lu, ^176m^Lu, ^174g^Lu, and ^174m^Lu. At shallow depths, where the deuteron energy is high, the production of isotopes is relatively low. As the deuteron energy decreases to approximately 40 MeV, corresponding to a depth of around 6 mm, the cross-section for producing ^174g+m^Lu increases (as shown in Figure 4a). At an energy of approximately 30 MeV, corresponding to a depth of about 7.5 mm, ^177^Lu production starts to increase. At an energy of around 20 MeV and a corresponding depth of 8.3 mm, the cross-section for ^176m^Lu production begins to rise. The ^174m^Lu cross-section reaches its peak at a depth of approximately 7.7 mm and then decreases to almost zero at a depth of 8.3 mm. For ^174g^Lu, its cross-section reaches its peak at a depth of around 7.9 mm and drops to almost zero at a depth of 8.5 mm. For both ^177^Lu and ^176m^Lu, they exhibit increasing cross-sections and peak at a depth of approximately 8.8 mm. The corresponding deuteron energy at this depth is approximately 13 MeV. Both ^177^Lu and ^176^Lu cross-sections decrease and approach zero at a depth of approximately 9 mm. As to be discussed shortly, the differences in in-depth profiles of different isotopes are important in maximizing the specific activity of ^177^Lu.

The depth-dependent cross-sections are used to calculate the quantity of isotope products at different depths for specific bombardment time and processing time. The change of isotopes per unit volume ( N ) is determined by the following equation [30],

$$dN=\left\{\begin{array}{c} \left(R-\lambda N\right)dt\;\;\;:0<t<t_{b}\\ -\lambda Ndt\;\;\;\;\;\;\;\;\;\;\;\;:t_{b}<t \end{array}\right.,$$

with the solution

$$N=\frac{R\left(1-e^{-\lambda t_{b}}\right)e^{-\lambda (t-t_{b})}}{\lambda }\;\;\;\;\;(for\;t>t_{b})$$

$$\lambda =0.693/t_{1/2}$$

$$R=\sigma N_{0}\Phi$$

where t is time, $t_{b}$ is the end of irradiation time, *R* is the reaction rate per unit volume, λ is the decay constant, $N_{0}$ denotes the atomic density (which is 2.422 × 10^22^ atoms/cm³ for Yb), and Φ the number of deuterons arriving on the surface per unit area per unit of time. For Φ , a value of 1 × 10^16^ deuterons/cm^2^/s is used, which is typical for a high-performance plasma source. Note that 1 × 10^16^ deuterons/cm²/s corresponds to 1.6 mA/cm^2^. The decay constants λ used in the calculations are based on the half-life times $t_{1/2}$ : 6.65d for Lu177g , 3.66h for Lu176m , 3.31y for Lu174g , and 142d for Lu174m .

**Figure 6:**
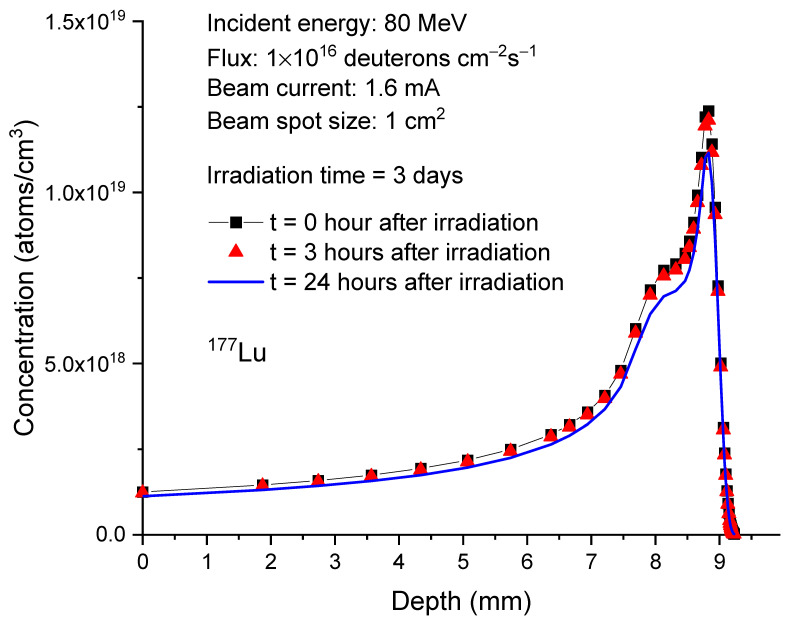
Concentrations of Lu177 as a function of depth in 80 MeV deuteron-irradiated Yb176 . The irradiation time is 3 days. For comparison, the post-irradiation processing times considered are 0 h, 3 h, and 24 h. The beam current is 1.6 mA. The beam spot size is 1 cm^2^. This corresponds to 1 × 10^16^ deuterons/cm²/s.

Figure 6 plots the calculated depth profiles of Lu177 in 80 MeV deuteron-irradiated ^176^Yb at three different time points: 0 h, 3 h, and 24 h after the completion of irradiation. The irradiation time is set to 3 days. Three hours after the irradiation, the concentration changes of Lu177 due to its decay are minimal. In 24 h after the irradiation, concentration decreases become apparent. However, the overall concentration changes are not significant. At the depth of peak concentration, 8.8 mm, the concentration of Lu177 decreases from 1.24 × 10^19^ atoms/cm^3^ to 1.21 × 10^19^ atoms/cm^3^ after three hours and further decreases to 1.12 × 10^19^ atoms/cm^3^ after 24 h.

**Figure 7:**
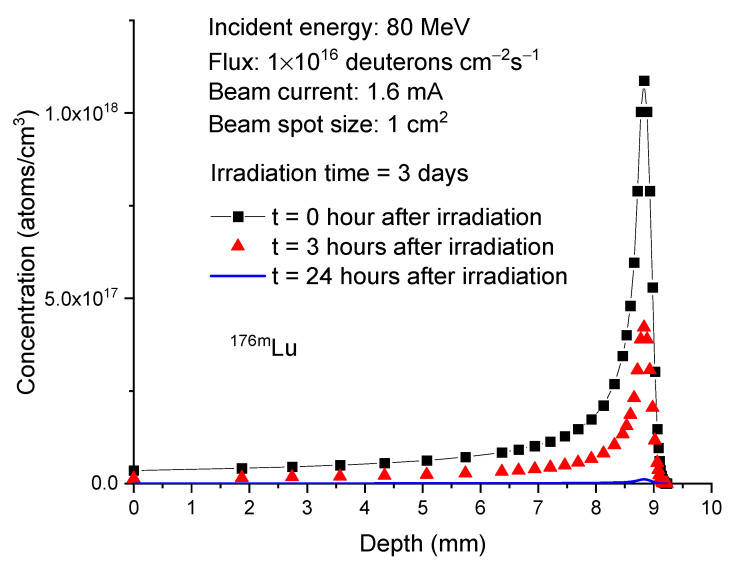
Concentrations of Lu176m as a function of depth in 80 MeV deuteron-irradiated Yb176 . The irradiation time is 3 days. The post-irradiation processing times are 0 h, 3 h, and 24 h. The beam current is 1.6 mA. The beam spot size is 1 cm^2^. This corresponds to 1 × 10^16^ deuterons/cm²/s.

**Figure 8:**
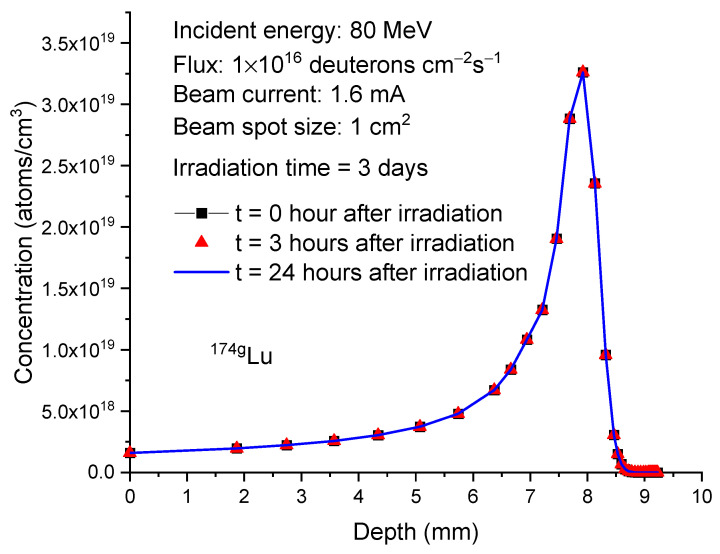
Concentrations of Lu174g as a function of depth in 80 MeV deuteron-irradiated Yb176 . The irradiation time is 3 days. The post-irradiation processing times are 0 h, 3 h, and 24 h. The beam current is 1.6 mA. The beam spot size is 1 cm^2^. This corresponds to 1 × 10^16^ deuterons/cm²/s.

**Figure 9:**
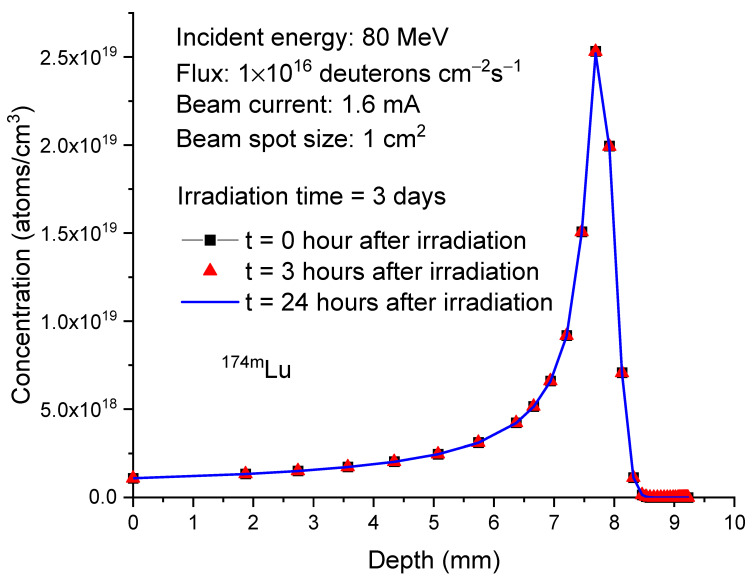
Concentrations of Lu174m as a function of depth in 80 MeV deuteron-irradiated Yb176 . The irradiation time is 3 days. The post-irradiation processing times are 0 h, 3 h, and 24 h. The beam current is 1.6 mA. The beam spot size is 1 cm^2^.

As a result of its much faster decay, the concentration change of ^176m^Lu differs significantly from that of ^177^Lu. Figure 7 plots the ^176m^Lu concentration profiles at the same time points as shown in Figure 6. At the end of irradiation, the peak concentration of ^176m^Lu is 1.09 × 10^18^ atoms/cm^3^. After 3 h, the concentration decreases to 4.22 × 10^17^ atoms/cm^3^ and further decreases to 1.16 × 10^16^ atoms/cm^3^ after 24 h. In contrast, both ^174g^Lu and ^174m^Lu exhibit negligible concentration changes, as shown in Figure 8 and Figure 9. This lack of changes is due to their relatively long half-lives, which are 3.31 years for ^174g^Lu and 142 days for ^174m^Lu. It is worth noting that the peak concentrations of both isotopes, at 2.5–3.5 × 10^19^/cm³, are higher than the peak concentration of ^177^Lu (about 1 × 10^19^/cm³). 

**Figure 10:**
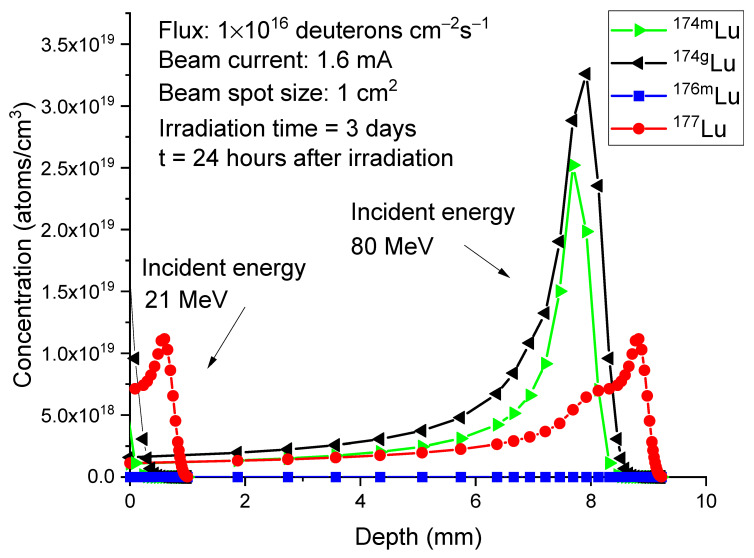
Concentration profiles of ^177^Lu (red circle), ^176m^Lu (blue square), ^174g^Lu (black triangle), and ^174m^Lu (green triangle) for the incident energies of 80 MeV and 40 MeV. The irradiation time is 3 days. The calculations correspond to 24 h after the irradiation. The beam current is 1.6 mA. The beam spot size is 1 cm^2^.

Considering the differences in concentration distributions and decay rates, we propose two methods to increase the specific activity of ^177^Lu. The first method involves reducing the deuteron beam energy to a value close to 21 MeV. This energy is high enough to activate the production of ^177^Lu but not high enough to produce significant amounts of ^174g^Lu and ^174m^Lu. Figure 10 compares the isotope profiles for an incident energy of 80 MeV and an incident energy of 21 MeV. The calculations correspond to a post-irradiation processing time of 24 h, with the irradiation time set to 3 days. In the case of 21 MeV, ^177^Lu reaches its peak concentration at a depth of 0.6 mm and drops to zero at approximately 1 mm depth. The unwanted isotope ^174g^Lu is present in small quantities near the surface and rapidly decreases to nearly zero at a depth of around 0.4 mm. The concentration of ^174m^Lu is located at even shallower depths and is negligible. Due to its rapid decay, the concentration of ^176m^Lu is reduced to a negligible level as well.

**Figure 11:**
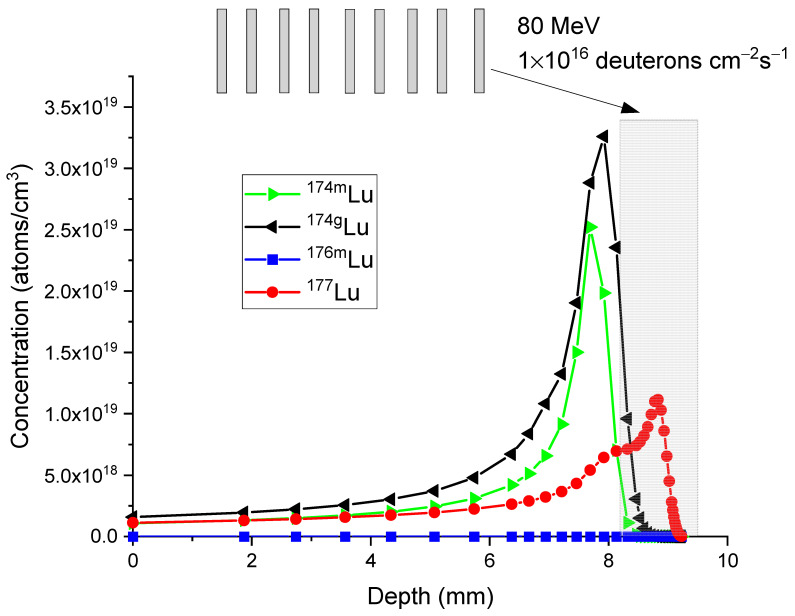
Concentration profiles of ^177^Lu (red circle), ^176m^Lu (blue square), ^174g^Lu (black triangle), and ^174m^Lu (green triangle) for the incident energies of 80 MeV in a stacked foil target configuration. The irradiation time is 3 days. The calculations correspond to 24 h after the irradiation. The beam current is 1.6 mA. The beam spot size is 1 cm^2^.

The second method involves the use of a stacked foil target. The stacked-foil technique is commonly employed to measure excitation functions of nuclear reactions involving light ions as projectiles [31,32,33]. In this technique, a target composed of multiple foils is used. In the present study, stacked foils are proposed to harvest isotopes at specific beam energies. Figure 11 illustrates the arrangement where ^176^Yb targets with approximately 1 mm thickness are stacked and exposed to deuteron beams. The thickness of the target foils is slightly adjusted to ensure that the last foil corresponds to a depth ranging from 8.22 mm to 9.22 mm. The gray colored box indicates the last foil, where the specific activity of ^177^Lu is maximized, and the unwanted isotopes are minimized.

Table 2 and Table 3 compare the daily isotope production from different depth regions: 8.22 mm to 9.22 mm (the last foil) and the entire depth range of 0 to 9.22 mm (all foils). The daily production is based on two rounds of irradiation, with each round lasting 12 h. For each round, the post-irradiation chemical separation time is 28 h. This condition arises after optimization to maximize specific activities. It does not represent the conditions to maximize radionuclidic purity nor the conditions optimized to balance both specific activity and radionuclidic purity. The conditions for different optimization needs will be discussed shortly. Table 2 presents the first optimization condition, prioritizing specific activity. The tables provide information on the activity, mass, specific activity, and radionuclidic purity of ^174m^Lu, ^174g^Lu, ^176m^Lu, and ^177^Lu.

In Table 2, which pertains to the last foil only, the activity of daily produced ^177^Lu is 304 GBq, and its specific activity is 3084 GBq/mg. Although the activity is relatively low, the specific activity is remarkably high. Note that the majority of reactor-based neutron irradiation produces ^177^Lu with a specific activity ranging from 740 to 1100 GBq/mg. In Table 3, which covers the entire depth region from 0 to 9.22 mm, the activity of ^177^Lu is 1119 GBq, with an average specific activity of 1022 GBq/mg. Even though the specific activity is lower compared to the previous cases, it is still comparable to the quality of reactor-produced isotopes.

**Figure 12:**
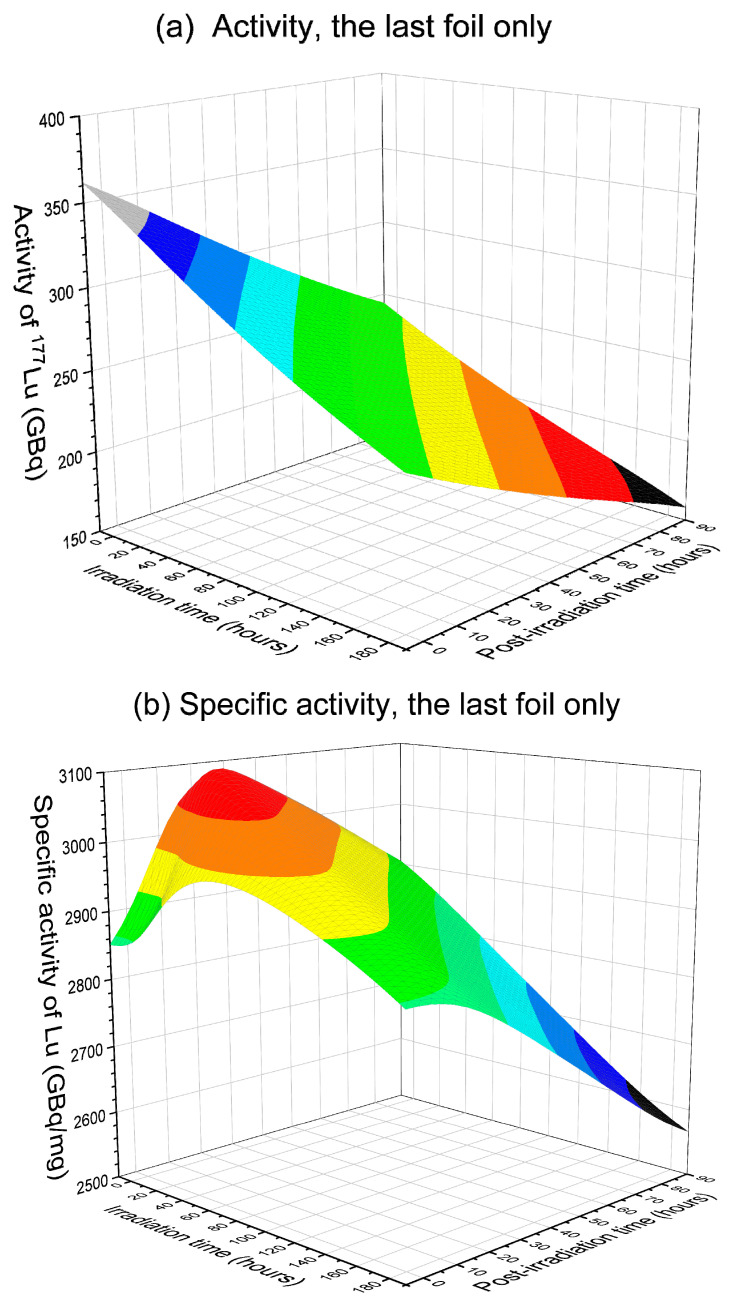

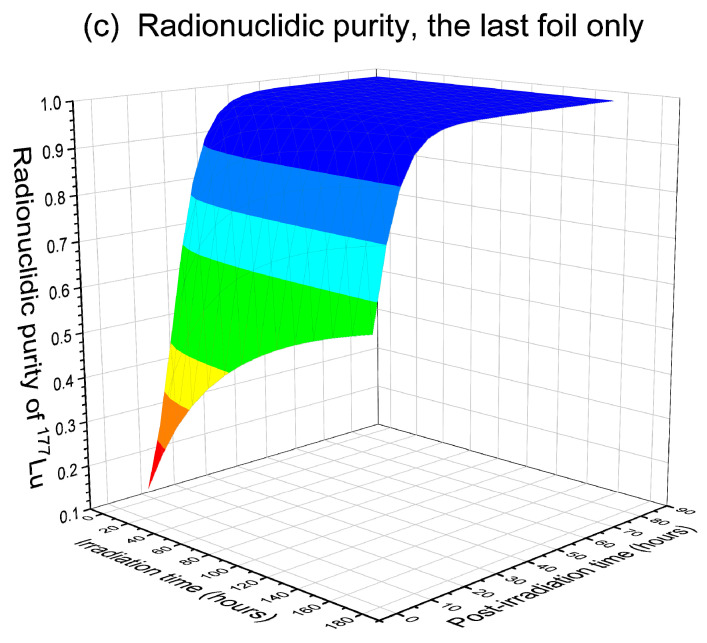
(**a**) Activity, (**b**) specific activity, and (**c**) radionuclidic purity of ^177^Lu collected from the last foil (corresponding to a depth from 8.22 mm to 9.22 mm) of ^176^Yb irradiated using 80 MeV deuterons. The x-axis represents the irradiation time, and the y-axis represents the post-irradiation processing time. All yields are normalized to six days of operation. The flux is 1 × 10^16^ deuterons/cm^2^/s, which is equivalent to a beam current of 1.6 mA over a beam spot size of 1 cm^2^.

**Figure 13:**
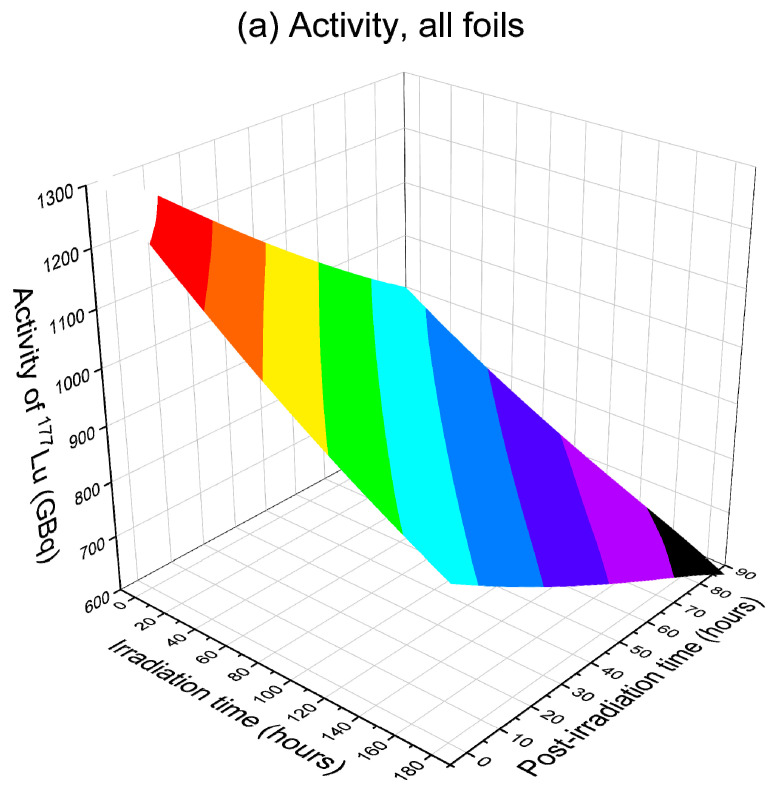

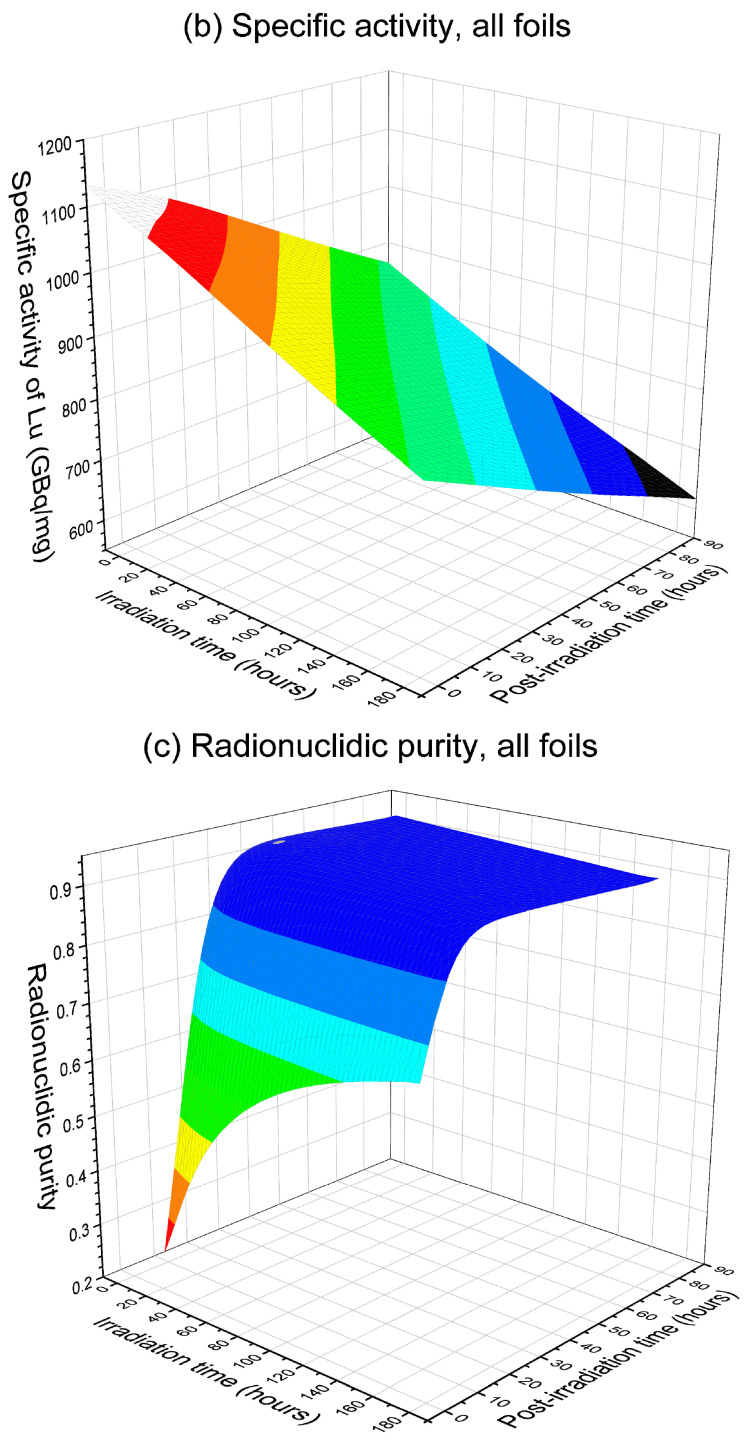
(**a**) Activity, (**b**) specific activity, and (**c**) radionuclidic purity of ^177^Lu collected from all foils (corresponding to a depth from 0 mm to 9.22 mm) of ^176^Yb irradiated using 80 MeV deuterons. The x-axis represents the irradiation time, and the y-axis represents the post-irradiation processing time. All yields are normalized to one day of operation. The flux is 1 × 10^16^ deuterons/cm^2^/s, which is equivalent to a beam current of 1.6 mA over a beam spot size of 1 cm^2^.

To maximize ^177^Lu production, both in terms of total activity and specific activity, calculations were performed to determine the optimized conditions. The calculations changed both the irradiation time and the post-irradiation processing time incrementally. Figure 12 and Figure 13 compare the isotope quality of the last foil and all foils. These plots emphasize the significance of selecting the appropriate processing time window. In the calculations, irradiation time changes using a step of 12 h, while post-irradiation processing time changes using a step of 4 h. All plots are normalized to one day of operation. If each batch requires a 12 h irradiation period, then one day of operation combines the yields from two batches. The post-irradiation processing start time is not limited to a duration shorter than one day. The calculation takes into account continuous operations, allowing the post-irradiation processing time to extend beyond one day if necessary.

Figure 12 provides a summary of the product quality of the last foil. All figures correspond to one day of operation (two batches per day). In Figure 12a, the total activity of ^177^Lu reaches the maximum of approximately 337 GBq/day for an irradiation time of 12 h and a post-irradiation processing time of 4 h. Increasing either the irradiation time or the post-irradiation processing time leads to a decrease in activity. Figure 12b plots the specific activity and exhibits the existence of a peak region. At a given irradiation time, the specific activity initially increases and then decreases with increasing post-irradiation processing time. The peak value is approximately 3071 GBq/mg. For maximizing specific activity, the optimal post-irradiation processing time is around 20 h for short irradiation. The optimal post-irradiation processing time slightly reduces to about 12 h for long irradiation. Prolonged irradiation and post-irradiation processing times result in a drop in specific activity, with the lowest value reaching approximately 2590 GBq/mg, representing the worst scenario in the mapping. However, an overall high specific activity can still be ensured.

In Figure 12c, the radionuclidic purity of the last foil is plotted. Unlike activity and specific activity, the radionuclidic purity approaches a saturated maximum of 99.6%. To achieve a high radionuclidic purity, an extended post-irradiation processing time is needed. By combining Figure 12a–c, an optimized time window can be identified. In summary, maximizing activity requires a short irradiation time and a short post-irradiation processing time, maximizing specific activity necessitates a post-irradiation processing time window of approximately 20 h, and maximizing radionuclidic purity calls for a prolonged post-irradiation processing time. A balanced condition can be achieved, such as selecting a 12 h irradiation time and a 28 h post-irradiation processing time, resulting in an activity of 304 GBq/day, a specific activity of 3084 GBq/mg, and a radionuclidic purity of 95.4%. As mentioned earlier, this is not the condition to maximize radionuclidic purity. The condition for maximizing radionuclidic purity will be discussed shortly.

Figure 13a–c plots the activity, specific activity, and radionuclidic purity for all foils (corresponding to a depth region of 0 to 9.22 mm). As mentioned earlier, including these foils containing a high concentration of ^174g^Lu and ^174m^Lu degrade the overall isotope quality. However, these foils can still be used in certain medical applications where low specific activity is acceptable. In Figure 13a, it is evident that including all foils significantly increases the total activity. The highest activity of ~1260 GBq/day can be achieved by minimizing both the irradiation time (12 h) and the post-irradiation processing time (4 h). Regarding specific activity, as shown in Figure 13b, the highest value of about 1100 GBq/mg can be obtained by minimizing the irradiation and post-irradiation processing time. Even in the worst-case scenario with a prolonged post-irradiation processing time exceeding 60 h, the specific activity remains at approximately 740 GBq/mg or above, which is comparable to typical products obtained via reactor irradiation. For radionuclidic purity, a prolonged post-irradiation processing time is favored, and the maximum value saturates at around 93%. 

Different from Figure 12b, which shows a post-irradiation processing time window for achieving maximum specific activity, there is no such window observed from Figure 13b. This discrepancy arises from the high sensitivity of specific activity to the changes of ^176m^Lu quantity in the last foil, given that ^176m^Lu and ^177^Lu are the predominant isotopes present, with ^176m^Lu having a rapid decay. In contrast, when all foils are considered, the mass changes contributed by ^176m^Lu are relatively insignificant due to the substantial contributions from ^174g^Lu and ^174m^Lu, which have almost negligible changes due to their relatively long half-lives. Consequently, the specific activity becomes less sensitive to changes in ^176m^Lu and more responsive to the activity changes of ^177^Lu itself.

Back to the approach of harvesting ^177^Lu from the last foil only, the obtained ^177^Lu under optimized conditions, as shown in Figure 12b, exhibits a remarkably high specific activity of about 3084 GBq/mg. This makes the process very attractive for hospital applications. This specific activity clearly surpasses the current achieved results using reactors. Table 4 provides a summary of the typical specific activities of ^177^Lu from different sources [13]. It is worth noting that 3084 GBq/mg is about 75% of the highest specific activity achievable in carrier-free ^177^Lu. The theoretically predicted maximum is 4104 GBq/mg [13]. 

Having all foils made of ^176^Yb is not a requirement in the stacked foil approach. Sacrificial materials of less expensive materials can be used in the other foils while reserving the last foil specifically for ^176^Yb. Alternatively, for isotope production other than ^177^Lu, different target materials can be used to replace the other foils. This flexibility is particularly valuable for isotopes that require a higher threshold energy for production.

The discussion above has assumed a ^176^Yb target with a purity level of 100%, which represents an ideal scenario. In reality, targets of a purity level of >99.6% are commercially available (i.e., from ISOFLEX USA, San Francisco, CA, USA). In commercially enriched ^176^Yb targets, impurity levels of other Yb isotopes decrease significantly as the mass numbers of Yb decrease [23]. Consequently, ^174^Yb is more abundant than other isotope impurities in ^176^Yb-enriched targets. The three unwanted products originating from ^174^Yb are ^174g+m^Lu and ^173^Lu. These isotopes have relatively long lifetimes compared to others, resulting in their minimal contribution to the radionuclide impurities of ^177^Lu.

To model the effect of purity, calculations were performed for the production and decay of ^177^Lu, ^174m^Lu, ^174g^Lu, ^176m^Lu, and ^173^Lu. The production calculations utilized cross-section data from Figure 4 and Figure 5, while the decay calculations were based on the half-life times provided in Table 1. Table 5 compares the activity, specific activity, and radionuclidic purity of ^177^Lu in ^176^Yb targets at purity levels of 100%, 99.6%, 99%, and 98%, respectively. All values correspond to daily production, assuming two rounds of irradiation per day with a 12 h irradiation time for each batch and a 28 h post-irradiation processing time for each batch. The conditions include an 80 MeV beam energy, 1.6 mA beam current, and a 1cm^2^ beam spot size. The comparison is made for collecting Lu from all foils (0 to 9.22 mm) and from the last foil (8.22 mm to 9.22 mm).

Table 5 shows that there is no significant difference when the purity is reduced from 100% to 99.6%. In the case of the last foil, the activity changes from 304 to 302 GBq and the specific activity changes from 3084 to 3052 GBq/mg. Even in the worst-case scenario of 98% purity, the activity is 335 GBq, and the specific activity is 2912 GBq/mg. These results suggest that a purity level of around 98% and above does not significantly degrade the quality of the final product. 

Using 99.6% purity as the example, Table 6 lists the activity, mass, and specific activity of each Lu isotope, giving further details about the impurity effect. Judged by the radionuclidic purity, the largest impurity effect comes from ^176m^Lu, at a value of 4.3%. ^176m^Lu has a specific activity of 137 GBq/mg. Although its mass is not the largest, its short half-life of 3.66 h makes its contribution large. On the other hand, ^173^Lu, as a unique product from ^174^Yb, has the smallest contribution in influencing ^177^Lu radionuclidic purity.

**Figure 14:**
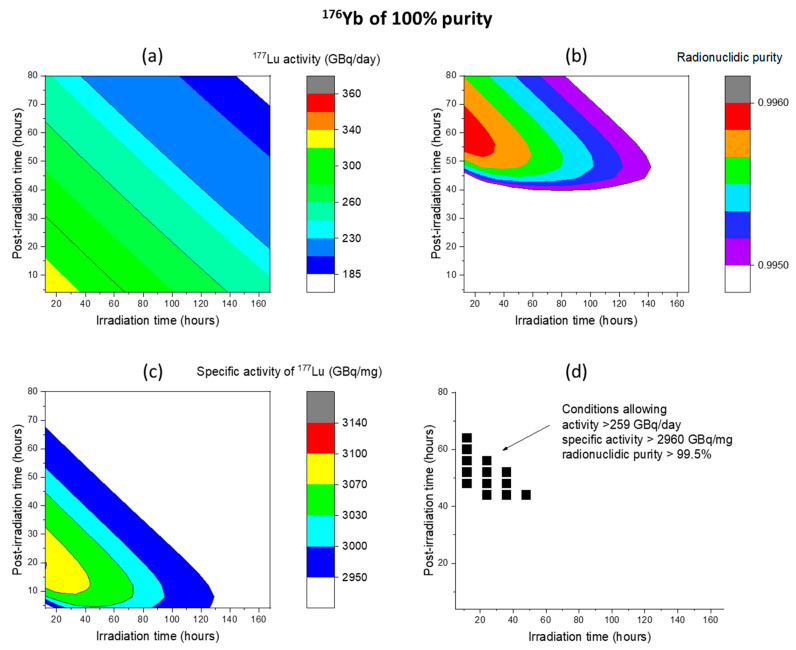
(**a**) Activity, (**b**) specific activity, (**c**) radionuclidic purity of ^177^Lu obtained from 80 MeV deuteron irradiation of ^176^Yb with 100% purity, and (**d**) processing time window capable of achieving activity > 259 GBq/day, specific activity > 2960 GBq/mg, and radionuclidic purity > 99.5%. Only the last foil is processed. The beam current is 1.6 mA, and the beam spot size is 1 cm^2^.

As one highlight of the present study, irradiation, and processing conditions are identified to provide the quality range that is of particular interest to the isotope production community. The most demanded quality is specific activity > 2960 GBq/mg and radionuclidic purity > 99.5%. Figure 14a–d are for ^176^Yb of 100% purity and for the last foil only (depth from 8.22 mm to 9.22 mm). Figure 14a maps the region where the activity exceeds 185 GBq/day, a condition that can be easily achieved based on the modeled conditions. The majority of regions surpass 259 GBq/day, except for a specific corner associated with significantly longer irradiation time and post-irradiation processing times. In Figure 14b, the plot showcases the region that surpasses 99.5% radionuclidic purity, which can be attained with post-irradiation processing times longer than 40 h. This region appears to be less sensitive to variations in irradiation times. Figure 14c presents the region of the specific activity exceeding 2960 GBq/mg. It shows a trade-off between irradiation time and post-irradiation processing times, whereby a longer irradiation time necessitates a shorter post-irradiation processing time. Figure 14d provides a summary of the region that satisfies the combined criteria of activity > 259 GBq/day, specific activity > 2960 GBq/mg, and radionuclidic purity > 99.5%. It represents the optimized conditions obtained by considering all restrictions. This region corresponds to irradiation times shorter than 60 h and post-irradiation processing times between 44 and 64 h.

**Figure 15:**
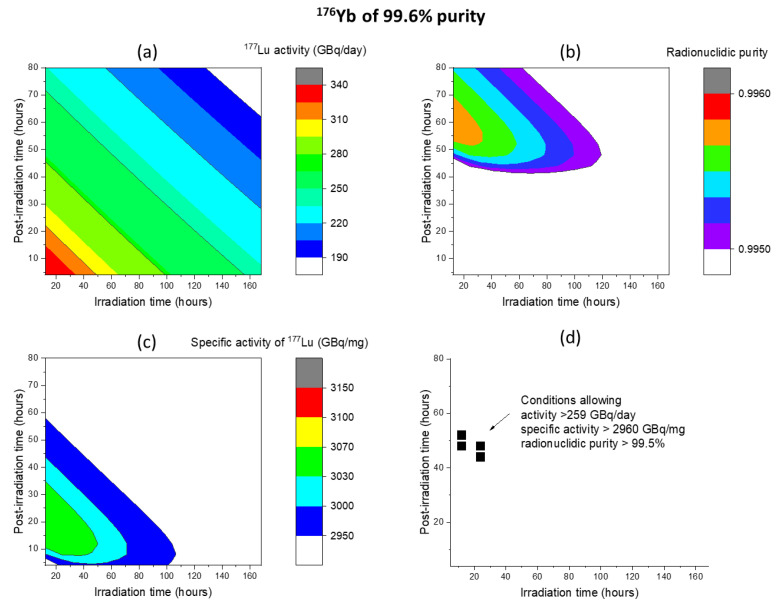
(**a**) Activity, (**b**) specific activity, (**c**) radionuclidic purity of ^177^Lu obtained from 80 MeV deuteron irradiation of ^176^Yb with 99.6% purity, and (**d**) processing time window capable of achieving activity > 259 GBq/day, specific activity > 2960 GBq/mg, and radionuclidic purity > 99.5%. Only the last foil is processed. The beam current is 1.6 mA, and the beam spot size is 1 cm^2^.

For a purity of 99.6%, Figure 15a–d plots the corresponding activity, specific activity, radionuclidic purity, and the optimized conditions for achieving activity > 259 GBq/day, specific activity > 2960 GBq/mg, and radionuclidic purity > 99.5%. These maps exhibit similarities to the case of 100% purity, with the expected difference that the regions are slightly smaller. The shrinking specific activity region and specific activity region impose further constraints on the conditions. As shown in Figure 15d, although conditions are narrowed down, a region still exists for attaining the required quality. The necessary irradiation time ranges from 12 to 24 h, while the post-irradiation processing time is around 48 h.

As pointed out in the introduction, the present study used 80 MeV as one example of high-energy LINAC facilities. However, the proposed multiple foil targets can be easily adjusted for other energies. The deuteron ions enter into the last foil at an energy of about 21 MeV. For other beam energies, as long as the foils prior to the last one can reduce beam energies down to 21 MeV, the product of the same quality can be obtained.

As shown in Figure 2, for deuterons reaching 21 MeV, deuterons penetrate about 4.7 mm for an initial beam energy of 60 MeV and about 1.9 mm for an initial beam energy of 40 MeV. As long as the foils ahead of the last one have a combined thickness matching these numbers, the same optimized conditions can be reached for the last foil.

Currently, isotope production is primarily dominated by reactor irradiation and cyclotrons. Most cyclotrons operate at relatively lower beam energies, typically around 40 MeV or lower. In contrast, LINACs are capable of delivering beam energies exceeding 40 MeV. LINACs can serve as versatile devices for isotope production, even for isotopes that favor lower beam energies, utilizing techniques like the one proposed in this study. It is important to note that typical beam currents for cyclotrons are around 100s μA, whereas LINACs can achieve beam currents of a few mA. For a high-energy LINAC, it is not ideal for Lu isotope production since much power is wasted if only the last foil is used. However, the design allows replacing these wasted foils with targets for producing other isotopes that require higher threshold (or optimized) energy.

## 3. Conclusions

By employing a simulation approach that combines particle transport and isotope production/decay simulations, the process of producing ^177^Lu via deuteron irradiation of ^176^Yb has been optimized, with a particular focus on maximizing the specific activity of ^177^Lu. Stacked foils have been proposed as the target for a linear accelerator operating at relatively high design energies. The selection of foils can be tailored to minimize the presence of ^174m^Lu and ^174g^Lu due to their distinct energy-dependent cross-sections. To minimize the production of ^176m^Lu, optimization of the post-irradiation processing time times has been investigated. Upon optimization, deuteron irradiation at 80 MeV and at a flux of 1 × 10^16^ deuterons/cm^2^/s (equivalent to a beam current of 1.6 mA over a beam spot size of 1 cm^2^) can result in daily production of 304 GBq of ^177^Lu, with a high specific activity of 3084 GBq/mg and a high radionuclidic purity of 95.4%. If specific activity and radionuclidic purity need to be balanced, there is an optimized condition that can obtain activity greater than 259 GBq/day, specific activity greater than 2960 GBq/mg, and radionuclidic purity greater than 99.5%.The study suggested the feasibility of utilizing a high energy, high current linear accelerator for the production of ^177^Lu at a specific activity level better than a typical reactor produced ^177^Lu.

## Figures and Tables

**Table 1:** Selected Lu isotopes and their decay characteristics [28,29].

Nuclide	Half-Life	Decay	Daughter Isotope
^169^Lu	34.06(5) h	β^+^	^169^Yb
^170^Lu	2.012(20) d	β^+^	^170^Yb
^170m^Lu	670(100) ms	IT	^170^Lu
^171^Lu	8.24(3) d	β^+^	^171^Yb
^171m^Lu	79(2) s	IT	^171^Lu
^172^Lu	6.70(3) d	β^+^	^172^Yb
^172m^Lu	3.7(5) min	IT	^172^Lu
^173^Lu	1.37(1) y	EC	^173^Yb
^174^Lu	3.31(5) y	β^+^	^174^Yb
^174m^Lu	142(2) d	IT (99.38%)	^174^Lu
		EC (0.62%)	^174^Yb
^176^Lu	38.5(7) × 10^9^ y	β^−^	^176^Hf
^176m^Lu	3.664(19) h	β^−^ (99.9%)	^176^Hf
		EC (0.095%)	^176^Yb
^177^Lu	6.6475(20) d	β^−^	^177^Hf

**Table 2:** The daily production of Lu isotopes in the depth region of 8.22 mm to 9.22 mm in 80 MeV deuteron-irradiated pure ^176^Yb. The beam current is 1.6 mA. The beam spot size is 1 cm^2^. The irradiation time is 12 h for each batch. The post-irradiation processing time is 28 h. This is the condition optimized to maximize specific activity. The conditions to maximize radionuclide activity and the conditions to balance both specific activity and radionuclide activity are different and will be separately discussed shortly.

	Activity (GBq)	Mass (mg)	Specific Activity (GBq/mg)	Radionuclidic Purity
^174m^Lu	0.555	0.003	5.92	0.2%
^174g^Lu	0.481	0.021	5.18	0.1%
^176m^Lu	13.7	7.02 × 10^−5^	139	4.3%
^177^Lu	304	0.074	3084	95.4%

**Table 3:** The daily production of Lu isotopes in the depth region of 0 mm to 9.22 mm. The irradiation condition is the same as in Table 2.

	Activity (GBq)	Mass (mg)	Specific Activity (GBq/mg)	Radionuclidic Purity
^174m^Lu	62.5	0.32	57	5.1%
^174g^Lu	11.5	0.50	10.4	0.9%
^176m^Lu	30.3	1.56 × 10^−4^	27.8	2.5%
^177^Lu	1119	0.27	1022	91.5%

**Table 4:** Summary of the specific activity ranges of ^177^Lu commercially available [13].

Suppliers	Specific Activity (GBq/mg)
Perkin Elmer, USA	~740
ORNL, USA	1850–2960
MURR, USA	925
MDS Nordion, Canada	1665
ITG, Garching, Germany	2960
IDB Holland BV	~740

**Table 5:** The daily production of ^177^Lu in ^176^Yb targets with purities of 100%, 99.6%, 99%, and 98%. Comparison was made for the depth region of 8.22 mm to 9.22 mm (the last foil) and the range of 0 mm to 9.22 mm (all foils) under 80 MeV deuteron irradiation. The beam current is 1.6 mA. The beam spot size is 1 cm^2^. The irradiation time for each batch is 12 h, and the post-irradiation processing time is 28 h as the optimized conditions. The condition is not optimized to maximize radionuclidic purity.

Starting ^176^Yb Purity	Depth Region	Activity (GBq) per Day	Mass (mg) per Day	Specific Activity (GBq/mg)	Radionuclidic Purity
100%	8.22 mm to 9.22 mm	304	0.074	3084	95.4%
	0 mm to 9.22 mm	1119	0.273	1022	91.5%
99.6%	8.22 mm to 9.22 mm	302	0.074	3052	95.4%
	0 mm to 9.22 mm	1115	0.272	1017	91.4%
99%	8.22 mm to 9.22 mm	301	0.073	3004	95.3%
	0 mm to 9.22 mm	1108	0.270	1008	91.4%
98%	8.22 mm to 9.22 mm	297	0.073	2912	95.3%
	0 mm to 9.22 mm	1097	0.267	994	91.3%

**Table 6:** The daily production of various Lu isotopes in the ^176^Yb target with a purity of 99.6%. Collection is limited to the last foil of the depth region of 8.22 mm to 9.22 mm. The beam current is 1.6 mA, and the beam spot size is 1 cm². The irradiation time for each batch is 12 h, and the post-irradiation processing time is 28 h as the optimized conditions. The parameters are optimized to maximize specific capacity. If radionuclidic purity needs to be maximized, a different condition is needed.

Nuclide	Activity (GBq) per Day	Mass (mg) per Day	Specific Activity (GBq/mg)	Radionuclidic Purity
^177^Lu	302	0.0737	3052	95.36%
^174m^Lu	0.555	0.003	5.74	0.17%
^174g^Lu	0.518	0.022	5.07	0.16%
^176m^Lu	13.6	0.00007	137	4.30%
^173^Lu	0.0296	0.0005	0.296	0.01%

## Data Availability

Data are available from the author on reasonable request.
